# Neurogenic Pulmonary Edema in a Patient with Traumatic Brain Injury

**DOI:** 10.5334/jbsr.3766

**Published:** 2024-11-07

**Authors:** Elyn Van Snick, Bart Ilsen, Steven Raeymaeckers

**Affiliations:** 1 Vrije Universiteit Brussel, Belgium; 2 UZ Brussel, Belgium

**Keywords:** pulmonary edema, non‑cardiogenic pulmonary edema, traumatic brain injury

## Abstract

*Teaching point:* Neurogenic pulmonary edema is a rare complication of cerebral trauma and should be considered in trauma patients presenting with pulmonary edema when no other cause is found.

## Case History

**Figure 1:**
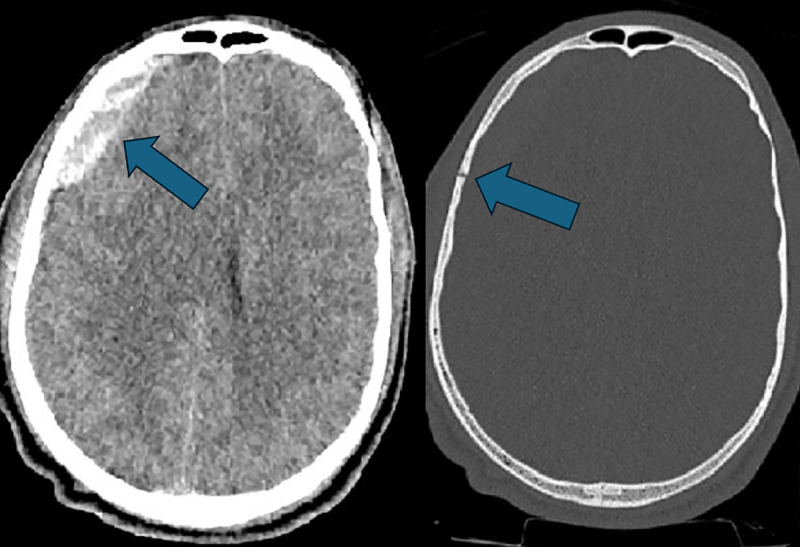
Head CT: skull fracture with underlying epidural hematoma.

A 16‑year‑old boy was admitted to the emergency department after being hit by a car while riding his bicycle. There was no loss of consciousness and Glascow Coma Scale was 15/15. The airway was clear, but oxygen saturation on pulse oximetry was only 91%. Whole body computed tomography (CT) scan revealed a skull fracture with underlying hyperacute epidural hematoma (Figure 1) and bilateral small areas of hemorrhagic contusion in the anterior temporal poles (not shown).

**Figure 2:**
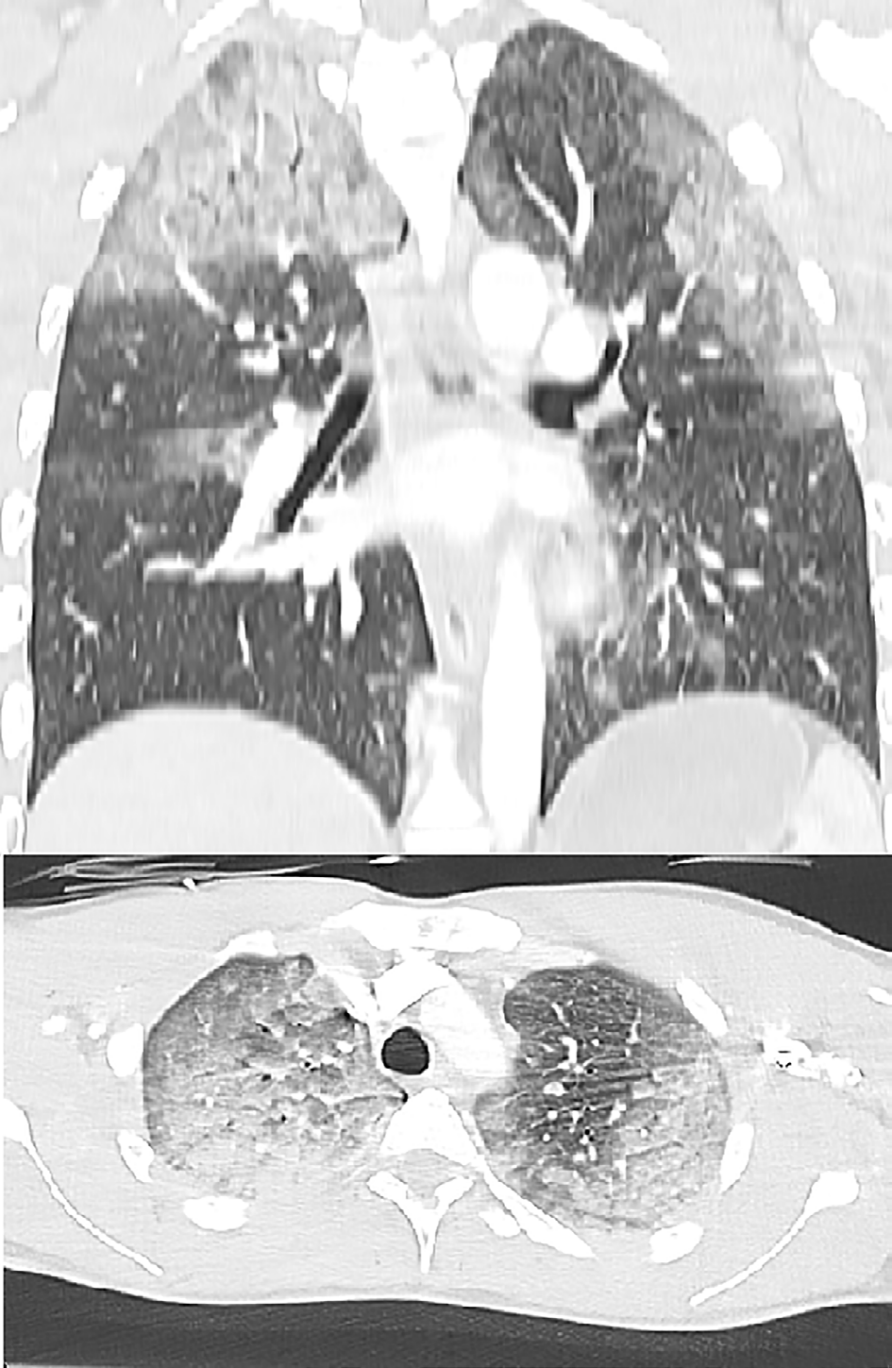
Chest CT: ground glass and consolidation, most pronounced in the upper lobes.

Chest CT (Figure 2) showed the presence of extensive areas of ground glass and consolidation in the dependent zones of all lung lobes, most pronounced in the upper lobes. There were no associated rib fractures nor pneumothorax.

Electrocardiogram and echocardiogram were normal and cardiac enzyme levels were not elevated.

**Figure 3:**
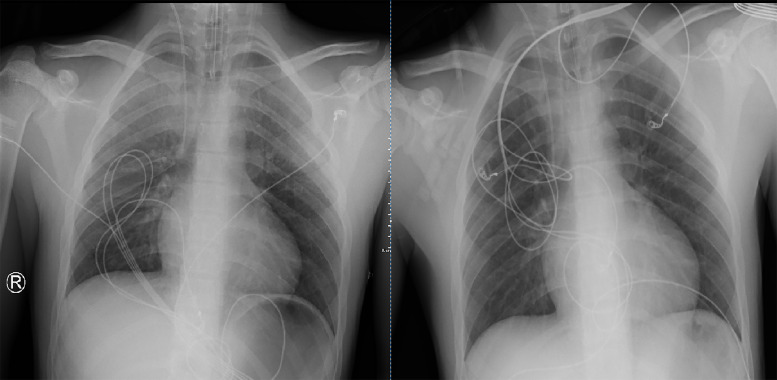
Chest X‑ray: rapid resolution of the opacities within a few days.

Follow‑up imaging with bedside lung X‑ray demonstrated rapid resolution of the lung parenchymal abnormalities within the next two days (Figure 3).

The pattern of lung parenchymal abnormalities and its rapid resolution over the next few days is consistent with neurogenic pulmonary edema.

## Discussion

Neurogenic pulmonary edema is a rare subtype of non‑cardiogenic pulmonary edema, caused by a neurological insult such as a seizure, traumatic brain injury, or a subarachnoid hemorrhage. The underlying pathophysiology is not fully understood, but it is likely to be the consequence of massive release of catecholamines by the sympathetic nervous system as a response to increased intracranial pressure, which in turn leads to increased hydrostatic pressure in the lungs and increased capillary permeability. It develops within minutes to hours after the neurological injury and spontaneously resolves within 48–72 h. Patients demonstrate symptoms of respiratory distress and might progress to respiratory failure.

Pulmonary imaging shows bilateral non‑specific airspace opacities with an apical predominance.

The condition can only be suggested by means of the association with a recent neurological insult and after excluding other causes.

Treatment depends on the patient’s condition from conservative to endotracheal intubation with mechanical ventilation. Diuretics and specific medication to reverse the effects of the sympathetic stimulation such as dobutamine or α‑adrenergic blockers may be used.

Mortality is high if patients are not appropriately diagnosed and treated [1].

## References

[r1] Tan CK, Lai CC. Neurogenic pulmonary edema. CMAJ. 2007;177(3):249–250. DOI: 10.1503/cmaj.061584. PMID: ; PMCID: . 17664447 PMC1930191

